# Physical activity, body composition and lipids changes in adolescents: analysis from the MyHeART Study

**DOI:** 10.1038/srep30544

**Published:** 2016-07-28

**Authors:** Hazreen Abdul Majid, Mohammadreza Amiri, Nahar Mohd Azmi, Tin Tin Su, Muhammad Yazid Jalaludin, Nabilla Al-Sadat

**Affiliations:** 1 Department of Social and Preventive Medicine, Faculty of Medicine, University of Malaya, 50603 Kuala Lumpur, Malaysia; 2 Department of Development Studies, Faculty of Economics and Administration, University of Malaya, 50603 Kuala Lumpur, Malaysia; 3 Dean’s Office, Faculty of Medicine, University of Malaya, 50603 Kuala Lumpur, Malaysia; 4 Centre for Population Health, University of Malaya, 50603 Kuala Lumpur, Malaysia; 5 Department of Paediatrics, Faculty of Medicine, University of Malaya, 50603 Kuala Lumpur, Malaysia

## Abstract

Insufficient physical activity and growing obesity levels among Malaysian adolescents are becoming a public health concern. Our study is to identify the trends of self-reported physical activity (PA) levels, blood lipid profiles, and body composition (BC) indices from a cohort of 820 adolescents. The self-reported PA was assessed using a validated Malay version of the PA Questionnaire for Older Children (PAQ-C). Fasting blood samples were collected to investigate their lipid profiles. Height, weight, waist and hip circumferences as well as body fat percentage were measured. The baseline and the first follow-up were conducted in 2012 and 2014, respectively. A downward trend in the PA level was seen in all categories with a significant reduction among all rural adolescents (*P* = 0.013) and more specifically, PA among girls residing in rural areas dropped significantly (*P* = 0.006). Either a significant reduction in high-density lipoprotein (HDL) or a significant increment in BC indices (i.e., body mass index [BMI], waist circumference [WC], hip circumference, and body fat percentage [BF %]) were seen in this group. Female adolescents experienced more body fat increment with the reduction of physical activity. If not intervened early, adolescents from rural areas may increase their risk of developing cardiovascular diseases earlier.

In adolescents, the level of physical activity (PA) is strongly correlated with cardiovascular fitness^1^, lipid profiles and body composition (BC) indices^2^. Being physically inactive, however, may lead to cardiovascular diseases (CVD)^1^,^2^,^3^. For instance, lower physical fitness due to insufficient levels and intensities of PA may lead to higher cardiometabolic risk in overweight/obese adolescents^3^. Cardiorespiratory fitness is important in the prevention of cardiovascular diseases^4^. In adolescents, PA determines the quality of lipid profiles - a study showed that lipid profiles in active overweight adolescents had a better quality (i.e., lower total cholesterol [TC], low-density lipoprotein cholesterol [LDL] and serum triglyceride [TG], while the high-density lipoprotein cholesterol [HDL] level was higher) than in non-active normal-weight children^2^. A recent study has shown that high body mass index during adolescence years is a very strong predictor of subsequent mortality in adults^5^.

In Malaysia, low PA among children is a major concern^6^, with about 64% of obese adolescents were physically inactive^7^. It has also been reported that obese adolescents in Malaysia had poor dietary intake^8^. In addition, reverse correlations between PA level and indicators of obesity (e.g., BMI, waist circumference [WC], and body-fat percentage [BF %]) among Malaysian adolescents suggested that the risk of obesity was greater among those who are physically inactive^7^. Therefore, immediate assessment of trends in PA level, BC indices, and lipid profiles seems vital in preventing the future negative health consequences^9^.

To the best of our knowledge, there is insufficient information about the trend of lipid profiles, BC indices and PA level among adolescents in South-East Asia and especially in Malaysia. Therefore, we aimed to assess the trends of these variables in a cohort that may help prospective prevention programs to take place among Malaysian adolescents in reducing future incidence of CVD.

## Results

**Figure 1:**
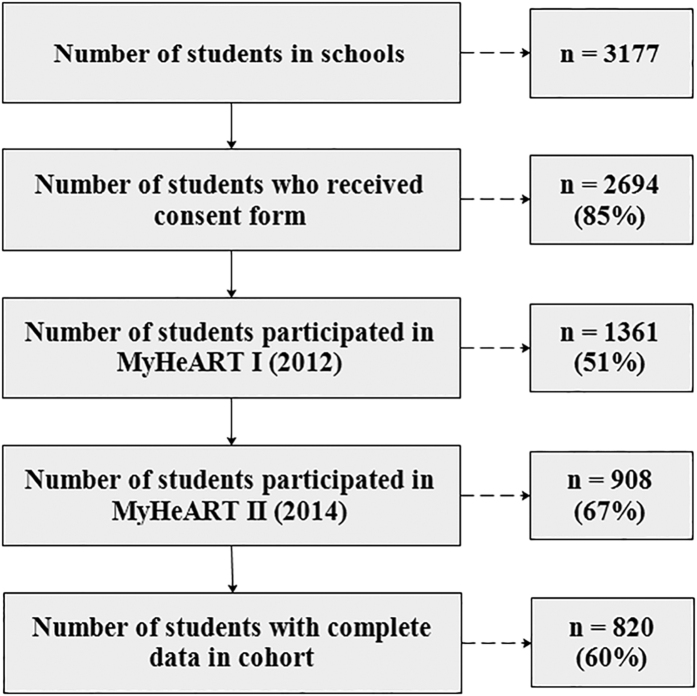
Number of students in each phase of the MyHeART study.

Out of 2694 students who received the invitation letter, 1,361 of them participated in the study (51%). Of these, 1,327 fasted prior to data collection and full data of them were available. The 2014 follow-up included 1,234 adolescents from which 917 were present at the baseline. Of these, 820 were fasted prior to data collection, participated in the second phase and all their data were available (Fig. 1). Therefore, the attrition rate (30%) was acceptable in cohort studies^10^.

In addition, there were no significant demographic differences found between participants and non-participants. In Table 1, the participant characteristics are presented. In the MyHeART study, the majority of the participants are girls (62.6%), Malays (80.5%), and urban residents (56.7%).

**Figure 2:**
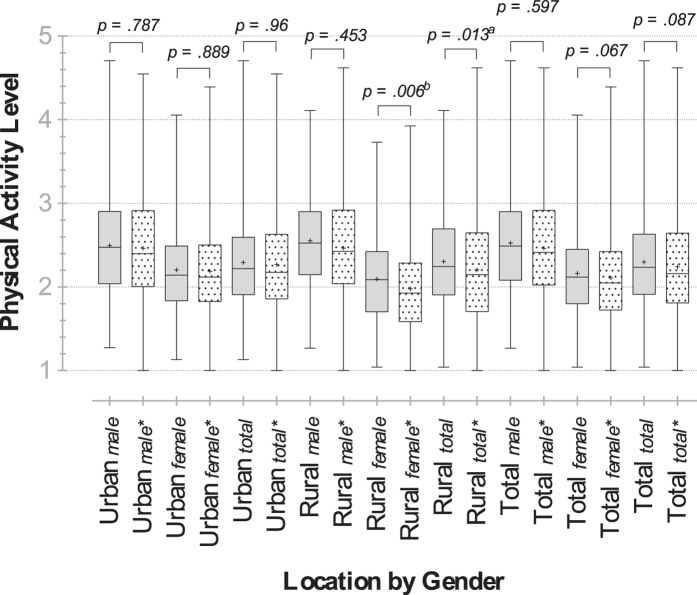
Physical activity level Box-Whisker plot for location by gender. Note: Wilcoxon matched-pairs signed-ranks test of median scores difference between 2012 and 2014 reported physical activity. The ‘**+**’ indicates mean, asterisks indicate values of the reported physical activity in 2014 while no asterisks indicated the same values in 2012. *p < 0.05; **p < *0.01* (Note: comparisons are just with the same location by gender category).

Figure 2 depicts the reported PA levels in the cohort and demonstrates the significant changes from the baseline to the first follow-up using the WSRT. In rural areas, the PA levels reduced by 5.4% reducing from *Mdn* = 2.24 (*IQR*: 1.90–2.70) in the baseline to 2.12 (1.70–2.64) in the first phase (*Z* = −2.471, *p* = 0.013). Interestingly while no significant change in the levels of PA was seen among boys, the girls in rural areas were sharply less active in 2014 (*Mdn*: 1.93; *IQR* = −2.28) than in 2012 (Mdn: 2.09; *IQR*: 1.72–2.43).

Table 2 illustrates the change in the blood lipid profiles in the cohort. Comparing results of the baseline and MyHeART I, in the total sample of adolescents we found a raise in LDL levels (*Z* = −2.752, *p* = 0.006), while the HDL levels dropped (*Z* = −3.682, *p* < 0.001). Contradictory results of lipid profiles between boys and girls were shown. While the boys had lower levels of TC (*Z* = −2.877, *p* = 0.004) and HDL level (Z = −6.609, *p* < 0.001), TC (*Z* = −3.367, *p* = 0.001), LDL (*Z* = −4.062, p < 0.001) and TG (*Z* = −12.261, *p* = 0.024) were increasing among the girls (see Table 2). However, girls who resided in urban areas experienced a raise in TC (*Z* = −3.242, *p* = 0.001) and a drop in TG levels (*Z* = −3.655, *p* = 0.001), whereas boys who resided in rural areas had lower TC levels comparing to the baseline (*Z* = −2.744, *p* = 0.006). In urban areas, while LDL level increased from 2.65 (2.22–3.13) in 2012 to 2.80 (2.3–3.2) in 2014 (*Z* = −4.135, *p* = 0.001), the HDL and TG levels decreased from 1.47 (1.30–1.64) and 0.86 (0.64–1.13) in 2012 to 1.4 (1.3–1.6) and 0.8 (0.6–1.1) in 2014 (*Z* = −2.847, *p* = 0.004; *Z* = −2.309, *p* = 0.021), respectively. However, in rural areas only HDL level significantly dropped from 1.44 (1.20–1.70) in 2012 to 1.40 (1.20–1.60) in 2014 (*Z* = −2.325, *p* = 0.02).

The descriptive analysis of the body composition indices in the cohort is presented in Table 3. Not surprisingly, at first glance, all the indices have significantly changed over the course of the cohort among the adolescents in this study who aged from 13 to 15 years old. However, whereas the BMI, WC, and hip circumference levels had increased significantly, BF percentage results were different between adolescents from different residential area where BF % in boys has dropped in total, urban, and rural; the girls’ fat composition level increased in the same stratifications. The WSRT indicated that the BF % among boys in the total sample decreased from 13.3 (6.9–26.2) in 2012 to 12.2 (7.5–22.5) in 2014, *Z* = −3.209, *p* = 0.001. Interestingly, this drop was only 1.3% in urban areas, while in rural areas it was 8.3%. In contrast, level of BF % had the highest increment among girls by 16.9, 18.0, and 16.0 percent in total, urban and rural areas respectively.

The correlation analysis (not presented here) illustrated the significant association between the change in the blood lipid profiles, BC indices, and the reported PA levels. Based on correlation results, the change in BMI was directly associated with the changes in LDL (*r* = 0.094, *p* = 0.008), TG (*r* = 0.136, *p* < 0.001), and TC (*r* = 0.108, *p* = 0.002), while inversely associated with the change in HDL (*r* = −0.101, *p* = 0.004). In addition, the change of WC was only significantly and directly associated with LDL (*r* = 0.166, *p* < 0.001) and TC (*r* = 0.001, *p* < 0.001), whereas the change of hip was only inversely correlated with HDL (*r* = 0.138, *p* < 0.001). Moreover, the change of BF % was directly associated with LDL (*r* = 0.144, *p* < 0.001) and TC (*r* = 0.177, *p* < 0.001) in the total sample.

## Discussion

In this study, we evaluated the existing trends of the self-reported PA, blood lipid profiles, and BC indices in Malaysian adolescents in a cohort starting from 2012. Then, we highlighted the significant correlations between the changes of the aforementioned variables. Identifying these trends, it is extremely important to address future interventions that may effectively reduce the obesity problem among this group^7^. Not only previous studies have illustrated that effective intervention programs on promoting PA in schools have resulted in improving general health by reducing BMI and WC levels, they have also shown to improve the academic performance of the adolescents^11^,^12^.

In the analysis of the cohort data for physical activity, we found no significant trend in the levels of PA in male adolescents as compared to the discovery of significant reducing trends of PA in total and rural girls, which are in line with previous findings in black girls in the US^13^. In line with our findings, Nelson, *et al*.^14^ found a reduction of PA level among girls from 5.9-4.9 hours/week from early to mid-adolescence and 5.1-3.5 hours/week from mid- to late adolescence. In Sweden, a sharp drop in PA level in adolescence was found from 1974 to 1995^15^. Although their study was conducted for over two decades, current study showed the same trend. The consequence of increased BMI and decreased PA level may result in worsening physical fitness in long term among adolescents^15^. These decreasing trends were partially due to increasing levels of sedentary lifestyle such as computer use in both girls and boys from 1999 to 2004. In addition, a British study found a remarkable reduction in PA in British adolescence between 1999 and 2004^16^, where girls (46%) had greater reduction compared to boys (23%). Previous studies on identifying trends in PA level among adolescents has neglected the environmental effects (e.g., urban vs. rural residential status). Therefore, not only did we present our results in both urban and rural areas, we also classified the residential status by gender. This was done with the intention to draw a detailed picture of the status of all the variables among gender and environmental characteristics that would certainly help future intervention to address the issues more accurately. A systematic review has highlighted several effective intervention policies to promote PA among adolescents^17^. van Sluijs, *et al*.^17^ concluded that school-based interventions should not only focus on the students but also the family or community. Multicomponent interventions are highly effective in PA promotion among school going adolescents. By incorporating physical activity promotion throughout the health care systems, it will encourage the subjects to be more active^18^,^19^.

In a previous study, a low-PA level is associated with lower HDL in normal weight adolescents but not overweight^20^. However, we were unable to identify any significant correlation between the change in PA level and any of the BC or lipid changes. Nonetheless, interventions in adolescents/children with dyslipidemia and/or abdominal obesity showed promising results as recreational PA cohort associated with nutritional counselling and lifestyle modifications represent an effective strategy to reduce risk markers^21^. These interventions are not only effective, but also cost effective^22^ in implementing them in schools to improve children’s quality of life and reduce lipid related risk to their health in adulthood. It is crucial to understand that by improving the cardiorespiratory fitness among the adolescents, it may delay the development of dyslipidemia^23^. Evidences also had shown the importance of physical activity and fitness in preventing CVD^24^,^25^.

Previous studies showed significant declines in TC level from 1980 to 1986^26^ and 1988 and 1994^27^ in the US adolescents, which are in contrast with our findings. Unlike our finding for TG level, which indicated no trend^28^, Viikari, *et al*.^26^ showed that in the US in a 6-year period the TG level increased from 0.79 to 0.84 mmol/l in 9–18 year old children and adolescents. This difference can be due to the difference between the level of PA and dietary intake between the two adolescent cohorts from different countries. Furthermore, we noted an increment in LDL level (*P* = *0.006*) and decreasing HDL level (*P* < *0.001*), while Viikari, *et al*.^26^ and Ford, *et al*.^28^ did not find such changes in their data. Reviewing the literature, we found that in the developed countries, the blood lipid profiles had alleviated sharply in adolescence between 1980 and 1992. For instance, a Finnish study found a decreased level of TC between 1980 and 1992 in adolescents who had the complete data in the longitudinal study of ‘*The Cardiovascular Risk in Young Finns Study*’^29^. There was a reduction of LDL and a significant drop of HDL level were found in the same period (19%), while an increment of 15% in TG levels from 1986 to 1992. These changes may have been the cause of the alteration in diet and a known trend toward increasing prevalence of obesity in both Finland and Europe^29^. Furthermore, obesity in these countries increased in a slower pace compared to Malaysia in recent years^30^,^31^.

Trends in BC indices implied significant increment in all variables except for BF % in boys (i.e., decreased in total as well as in urban and rural areas). The BF % change per annum in Malaysia is much alarming in comparison to a previous study conducted in Spanish adolescents that found 0.17%/year between 1995 to 2002^32^, while we showed 1.48%/year in total, 1.65%/year in rural, and 1.5%/year in urban resident adolescents. Our findings of BMI increment in boys is consistent with studies by Porkka, *et al*.^29^ and Westerstahl, *et al*.^15^ who found an increment in BMI levels from 1980 to 1992 in Finnish adolescents and from 1974 to 1995 in Swedish adolescents, respectively. However, while there is inconsistency in girls’ trend of BMI with Ogden, *et al*.^33^, as they found this trend only in boys aged 12 through 19 but not girls, we discovered a significant increment of BMI among female adolescents as total and from urban and rural residents.

This study is the first adolescent cohort study in Malaysia assessing the trend of physical activity level, body composition, and blood lipid profiles in Malaysian adolescents. Another strength of this study was the blood profiles collected which significantly enhances the accuracy of the values of the blood lipid profiles. However, the limitation of this study was the calculation of PA derived from self-reported values by students that may affect the validity of the PA scores. However, PAQ-C is a validated tool that has good internal consistency and acceptable validity in Malaysia^34^. PAQ-C assesses the level of PA and not the individual energy expenditure.

Adolescents in the rural areas, especially females, experienced worsening PA level, blood lipid profiles and body compositions. Interventions at schools are crucial with the support of parents and strong policies by the government to ensure a healthy nation, for today and the future.

## Methods

The description of the Malaysian Health and Adolescents longitudinal Research Team (MyHeART) study was reported elsewhere^35^. Briefly, the MyHeART study is a prospective cohort study of a representative sample of Malaysian adolescents who reside in Peninsular Malaysia. The data used in the current paper covered the MyHeART’s baseline (conducted in 2012) and the first follow-up (i.e., MyHeART I: conducted in 2014). At baseline, participants were 13-year-old schoolchildren studying in first year of public secondary schools (Secondary 1); while in MyHeART I, they were studying in Secondary 3 of the same school type. The inclusion criteria were that students must be able to read and write in Malay (Malaysia’s national language) and must fast overnight prior to data collection.

The MyHeART study was conducted in the Federal Territory of Kuala Lumpur (as a metropolitan area) and Selangor (Central region), and Perak (Northern region) states located in the Peninsular Malaysia. We selected these states purposively as per discussions held with the Ministry of Education of Malaysia.

### Sampling procedure

We applied a two-stage cluster sampling method. In the first stage, we randomly selected 15 secondary schools from a list of all secondary schools provided by the Ministry of Education. Then, in the second stage, all students attending Secondary 1 in the selected schools were invited to participate in the study.

### Measurements

#### Blood lipid profiles

Participants were asked to fast or stop taking food, and calorie contained food/drinks for at least ten hours before data collection for the baseline study and two years later during the follow-up. At both baseline and follow-up, a total of 15 ml of fasting blood was withdrawn from each participant at baseline and two years later during the follow-up cohort. The samples were sent to a certified International Organization for Standardization (ISO) hospital pathology lab for analysis. The samples were temporarily stored at four degrees Celsius in a cool box immediately after the blood had been withdrawn to preserve levels of markers that are sensitive to degradation due to increase in temperatures. All samples were processed in the field laboratories in the states. Samples were spun and stored as serum and divided into several aliquots of 0.5 ml of serum. The blood lipid profiles such as the high-density lipoprotein cholesterol (HDL), low-density lipoprotein cholesterol (LDL), triglycerides (TG), and the total cholesterol (TG) levels were measured at the field laboratories using Advia Chemistry, Siemens, Germany. All the aliquots for future lab analysis were stored at a temperature of 80 degree Celsius freezers at the University of Malaya (UM) laboratories. The same blood measurements in this cohort were repeated in the second phase of the cohort.

#### Anthropometric measurement

Height was measured without socks and shoes using a calibrated vertical Seca Portable 217 Stadiometer (Seca, United Kingdom), and rounded to the nearest millimeter. Weight was measured with light clothing using a Seca 813 digital electronic weighing scale (Seca, United Kingdom), and rounded to the nearest decimal fraction of a kilogram. Body mass index was calculated as weight in kilograms divided by height-squared in meters. Body-fat composition was estimated with bioelectrical impedance using a Tanita portable scale (SC- 240, Body Composition Analyzer, Tanita Europe B.V., The Netherlands). The Body Composition Analyzer SC–240 has acceptable accuracy compared to the dual-energy X-ray absorptiometry in white and African-American adolescents^36^. Waist circumference was measured with a non-elastic Seca measuring tape (Seca 201, Seca, UK), to the nearest millimeter. Waist circumference measurement was done at the natural waist which is the midway between the lowest rib margin (tenth rib) and highest point of the iliac crest with the tape around the body in horizontal position^37^. Trained research assistants did all the measurements.

#### Self-reported physical activity assessment

Self-reported physical activity levels were assessed using a validated Malay version of the Physical Activity Questionnaire for Older Children (PAQ-C) which has good internal consistency and acceptable validity^38^. There were 10 items in the PAQ-C that captured the level of physical activity in the last 7 days. The first item included the type and frequency of sports and/or dance activities the adolescents engaged in during the past seven days. The second to eighth items in the questionnaire assessed the activity of the adolescents during physical education (PE) classes, recess, lunchtime, right after school, evenings, weekend and leisure time. The answers to items 2 to 8 used a five-point Likert scale [one (lowest) to five (highest)]. Item nine included the frequency of participating in daily physical activity in the previous week. Item 10 asked the adolescents to report any unusual activities during the previous week, which have not already been recorded.

### Participation

The sample size was calculated using the following formula: n = (z^2^ × p × q/r × e^2^) × design effect, where z = 1.96 (i.e., standard normal deviate at 5% level for two-tailed test), p = estimated prevalence, q = (1–p), r = response rate and e was the precision level. We used the estimated prevalence of adolescent students who smoked in school^35^. The resulting sample number was about 1,500 participants.

We used computer generated random numbers to select fifteen schools that included 2,694 students. Information and consent forms were sent to parents or guardians of the students. The willingness and acceptance to participate in the study was confirmed when the written and signed consent form was received. In total, 1,361 (51%) adolescents participated in the baseline of this study from which about 820 (61%) were present at the follow-up (see Fig. 1). The attrition rate in this cohort from the baseline to the first follow up was about 39%. This attrition was likely because adolescents in Malaysia are highly dependent on their parents/guardians’ decisions. The majority of the non-responders did not attend the follow up study due to refusal from their parents/guardians who did not accept the consent forms both for baseline and the follow-up. In addition, the national examination to enter upper secondary grades (i.e., called PT3 in Malaysia) was due to be held at the end of the year when students were 15 years old in 2014.

### Ethics

The Ethics committee, University Malaya Medical Centre (Ref. no. 896.34), approved this study. Participation in the study was voluntary and written informed consent was obtained from the parents or guardian as well as the participants. The methods were carried out in accordance with the approved guidelines.

### Statistics

All data were analyzed using IBM SPSS Statistics V. 22 (Chicago, US). Descriptive and bivariate analyses were done as preliminary data analysis. As variables were not following Normal or Gaussian distributions, we presented them as Median (*Mdn*) and the Inter-Quartile Range (*IQR*: 25^*th*^–75^*th*^ *percentile*). In addition, we employed the Wilcoxon paired-samples signed-ranks Test (WSRT) to capture the significant changes in variables (i.e., blood lipid profiles, anthropometric measurements, and the reported physical activity) comparing to the baseline (in 2012). Finally, using bivariate Spearman correlation we analyzed whether there is any associations existed between the percentage change (
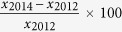
) between blood profiles, anthropometric indices, and the reported physical activities. The cut-off point of less than 0.05 was considered to mark the significant correlation coefficients.

## Additional Information

**How to cite this article**: Majid, H. A. *et al*. Physical activity, body composition and lipids changes in adolescents: analysis from the MyHeART Study. *Sci. Rep.* **6**, 30544; doi: 10.1038/srep30544 (2016).

## Figures and Tables

**Table 1:** Sample characteristics (n = 820).

	Number (%)
Gender	
*Male*	307 (37.4)
*Female*	513 (62.6)
Ethnicity	
*Malay*	660 (80.5)
*Chinese*	59 (7.2)
*Indian*	72 (8.8)
*Others*	29 (3.5)
Location	
*Urban*	465 (56.7)
*Rural*	355 (43.3)

**Table 2:** Test of median difference between lipid profile levels in cohort (n = 820).

			2012	2014	Wilcoxon signed ranks test
			Median (IQR^d^)	Median (IQR)	Z^a^ (p)
**Total**	**Total**	**LDL**^1^	2.69 (2.25–3.16)	2.7 (2.3–3.3)	−2.752^b^ (0.006)
		**HDL**^2^	1.45 (1.27–1.69)	1.4 (1.2–1.6)	−3.682^c^ (P < 0.001)
		**TG**^3^	0.82 (0.61–1.08)	0.8 (0.6–1.1)	−0.607^c^ (0.544)
		**TC**^4^	4.59 (4.1–5.16)	4.6 (4.1–5.2)	−0.897^b^ (0.37)
	**Male**	**LDL**	2.62 (2.18–3.07)	2.5 (2.1–3)	−0.785^b^ (0.433)
		**HDL**	1.41 (1.21–1.68)	1.3 (1.2–1.5)	−6.609^b^ (p < 0.001)
		**TG**	0.77 (0.59–1.04)	0.8 (0.6–1.1)	−1.906^c^ (0.057)
		**TC**	4.46 (4.00–5.00)	4.3 (3.8–5)	−2.877^b^ (0.004)
	**Female**	**LDL**	2.74 (2.32–3.25)	2.8 (2.4–3.3)	−4.062^c^ (p < 0.001)
		**HDL**	1.48 (1.30–1.70)	1.5 (1.3–1.6)	−0.559^c^ (0.576)
		**TG**	0.86 (0.62–1.10)	0.8 (0.6–1.1)	−2.261^b^ (0.024)
		**TC**	4.6 (4.17–5.20)	4.7 (4.3–5.3)	−3.367^c^ (p = 0.001)
**Urban**	**Total**	**LDL**	2.65 (2.22–3.13)	2.8 (2.3–3.2)	−4.135^b^ (p < 0.001)
		**HDL**	1.47 (1.3–1.64)	1.4 (1.3–1.6)	−2.847^c^ (0.004)
		**TG**	0.86 (0.64–1.13)	0.8 (0.6–1.1)	−2.309^c^ (0.021)
		**TC**	4.5 (4.10–5.10)	4.6 (4.1–5.2)	−1.906^b^ (0.057)
	**Male**	**LDL**	2.62 (2.12–3.05)	2.5 (2.1–3.1)	−0.043^c^ (0.965)
		**HDL**	1.45 (1.30–1.60)	1.3 (1.2–1.5)	−4.320^c^ (p < 0.001)
		**TG**	0.79 (0.62–1.08)	0.8 (0.6–1.2)	−1.164^b^ (0.245)
		**TC**	4.42 (4.10–4.94)	4.4 (3.8–5)	−1.587^c^ (0.113)
	**Female**	**LDL**	2.66 (2.25–3.16)	2.8 (2.4–3.3)	−4.935^b^ (p < 0.001)
		**HDL**	1.48 (1.30–1.67)	1.5 (1.3–1.6)	−0.612^c^ (0.54)
		**TG**	0.9 (0.66–1.17)	0.8 (0.6–1.1)	−3.655^c^ (p < 0.001)
		**TC**	4.6 (4.10–5.13)	4.7 (4.2–5.2)	−3.242^b^ (0.001)
**Rural**	**Total**	**LDL**	2.75 (2.30–3.28)	2.7 (2.3–3.3)	−0.643^c^ (0.52)
		**HDL**	1.44 (1.20–1.70)	1.4 (1.2–1.6)	−2.325^c^ (0.02)
		**TG**	0.77 (0.58–1.01)	0.8 (0.6–1.1)	−1.801^b^ (0.072)
		**TC**	4.63 (4.08–5.21)	4.5 (4–5.2)	−0.851^c^ (0.395)
	**Male**	**LDL**	2.64 (2.20–3.10)	2.5 (2.1–3)	−1.175^c^ (0.24)
		**HDL**	1.4 (1.19–1.70)	1.3 (1.1–1.5)	−5.092^c^ (p < 0.001)
		**TG**	0.77 (0.58–1.01)	0.8 (0.6–1.1)	−1.200^b^ (0.23)
		**TC**	4.5 (3.95–5.00)	4.3 (3.8–5)	−2.744^c^ (0.006)
	**Female**	**LDL**	2.89 (2.42–3.40)	2.9 (2.4–3.4)	−0.210^b^ (0.833)
		**HDL**	1.48 (1.23–1.70)	1.5 (1.3–1.7)	−1.618^b^ (0.106)
		**TG**	0.78 (0.58–1.03)	0.8 (0.6–1)	−1.189^b^ (0.234)
		**TC**	4.72 (4.20–5.36)	4.8 (4.3–5.4)	−1.293^b^ (0.196)

Note: 1. **LDL:** Low-density lipoprotein cholesterol; 2. **HDL:** High-density lipoprotein cholesterol; 3. **TG:** Triglyceride; 4. **TC:** Total cholesterol.

^a^Wilcoxon Sign-ranks Test.

^b^Based on negative ranks.

^c^Based on positive ranks.

^d^IQR: Interquartile range.

**Table 3:** Test of median difference between body composition indices in cohort (n = 820).

			2012	2014	Wilcoxon Signed Ranks Test
			Median (IQR^d^)	Median (IQR)	Z^a^ (p)
**Total**	**Total**	**BMI**^1^	18.54 (16.27–21.93)	19.87 (17.72–23.65)	−19.354^b^ (p < 0.001)
		**WC**^2^	65.75 (60.5–75)	69 (63.7–78)	−14.063^b^ (p < 0.001)
		**Hip**^3^	84 (78–92)	89.5 (84–96.58)	−21.919^b^ (p < 0.001)
		**BF%**^4^	21.45 (13.83–31.1)	24.4 (16.85–32.2)	−9.335^b^ (p < 0.001)
	**Male**	**BMI**	18.17 (15.94–21.62)	19.29 (17.21–23)	−10.883^c^ (p < 0.001)
		**WC**	65 (61–77)	68.5 (63.7–80)	−9.579^c^ (p < 0.001)
		**Hip**	80 (75–89)	86.5 (81.6–95)	−13.716^c^ (p < 0.001)
		**BF%**	13.3 (6.9–26.2)	12.2 (7.5–22.5)	−3.209^b^ (p = 0.001)
	**Female**	**BMI**	18.76 (16.63–22.14)	20.26 (18.02–23.93)	−16.031^c^ (p < 0.001)
		**WC**	66 (60–74)	69 (63.55–77)	−10.465^c^ (p < 0.001)
		**Hip**	86 (80–93)	90.5 (86–98)	−17.059^c^ (p < 0.001)
		**BF%**	23.7 (17.65–32.85)	27.7 (22.8–34.5)	−13.887^b^ (p < 0.001)
**Urban**	**Total**	**BMI**	18.61 (16.62–21.94)	19.92 (17.86–23.38)	−14.287^b^ (p < 0.001)
		**WC**	66 (61–75)	70 (64.7–78.9)	−10.951^b^ (p < 0.001)
		**Hip**	86 (80–92.5)	90.5 (85–97)	−16.239^b^ (p < 0.001)
		**BF%**	21.9 (15.5–31.2)	24.9 (19.3–32.2)	−7.677^b^ (p < 0.001)
	**Male**	**BMI**	18.57 (16.52–21.62)	19.67 (17.44–23)	−7.426^b^ (p < 0.001)
		**WC**	68 (62–78)	70.1 (64.7–80)	−5.793^b^ (p < 0.001)
		**Hip**	83 (78–90)	87.8 (82.6–95.7)	−9.394^b^ (p < 0.001)
		**BF%**	15 (8–25.5)	14.8 (8.7–22.4)	−2.030^c^ (0.042)
	**Female**	**BMI**	18.67 (16.63–22.19)	20.06 (18.03–23.67)	−12.153^b^ (p < 0.001)
		**WC**	66 (60.88–74)	70 (64.38–78)	−9.291^b^ (p < 0.001)
		**Hip**	86.25 (81–94)	91.55 (86.5–98.05)	−13.229^b^ (p < 0.001)
		**BF%**	23.3 (17.68–33.98)	27.5 (22.68–34.33)	−10.466^b^ (p < 0.001)
**Rural**	**Total**	**BMI**	18.41 (15.96–21.71)	19.69 (17.43–24.12)	−14.287^b^ (p < 0.001)
		**WC**	64 (60–75)	68.3 (62.85–76.1)	−10.951^b^ (p < 0.001)
		**Hip**	81 (76–90.3)	87.5 (82.8–96)	−16.239^b^ (p < 0.001)
		**BF%**	20.7 (11.8–31.05)	24 (11.9–32.2)	−7.677^b^ (p < 0.001)
	**Male**	**BMI**	17.53 (15.78–21.74)	18.73 (17.07–23.16)	−7.745^b^ (p < 0.001)
		**WC**	64 (60–75)	67.7 (63.2–83.8)	−7.405^b^ (p < 0.001)
		**Hip**	78 (74–87)	85.3 (81.1–92)	−9.775^b^ (p < 0.001)
		**BF%**	12 (6.1–28)	11 (6.7–24.1)	−2.606^c^ (0.009)
	**Female**	**BMI**	18.88 (16.45–21.67)	20.47 (17.99–24.26)	−10.465^b^ (p < 0.001)
		**WC**	65 (60–74)	68.5 (62.2–75.5)	−4.954^b^ (p < 0.001)
		**Hip**	84 (78–92)	89.1 (84.5–97)	−10.795^b^ (p < 0.001)
		**BF%**	24.3 (17.6–32.5)	28.2 (22.9–35)	−9.171^b^ (p < 0.001)

Note: 1. **BMI:** Body mass index; 2. **WC:** Waist Circumference; 3. **Hip:** Hip circumference; 4. **BF%:** Body fat percentage.

^a^Wilcoxon Sign-ranks Test.

^b^Based on negative ranks.

^c^Based on positive ranks.

^d^IQR: Interquartile range.
